# A model for transforming the digital landscape of medical universities

**DOI:** 10.6026/973206300211895

**Published:** 2025-07-31

**Authors:** Angel Rajan Singh, Amitesh Khare, Arun Verma

**Affiliations:** 1 Department of Hospital Administration, All India Institute of Medical Sciences (AIIMS), New Delhi, India

**Keywords:** Hospital information system, digital transformation of hospitals, cybersecurity

## Abstract

As healthcare systems worldwide grapple with the challenges of digital transformation, the convergence of technology and clinical excellence has become a universal priority. However, the historical and partial adoption of digital services in hospitals has been a significant barrier due to the disjointed nature of technology adoption, resulting in numerous solutions operating in silos with interoperability concerns that negatively affect patient care and effectiveness. Therefore, it is of interest to introduce a comprehensive and adaptable 5-Pillar Model designed to guide hospitals and medical universities, regardless of their geographic or economic context, toward achieving quality-driven care through strategic digital transformation. The model emphasizes five critical pillars such as reliable network infrastructure, microservices-based applications with a federated architecture, hybrid cloud-based data centers with disaster recovery, robust cybersecurity measures and governance for sustained quality assurance of digital systems.

## Background:

Digital transformation of hospitals [1, 2] refers to fundamental rewiring of how it operates. It involves the integration of digital technologies into all areas of a hospital's operations, with the goal of improving patient care, enhancing the overall efficiency of academics & healthcare delivery and reducing costs. This transformation often involves implementing electronic health records (EHRs), telemedicine, digital imaging and other technologies to streamline processes, increase patient engagement and facilitate data-driven decision-making [3, 4]. The goal of digital transformation in hospitals and healthcare is to provide better outcomes and experiences for patients, while also supporting the continued growth and advancement of the institutions. Medical Universities often represent the apex of the tertiary healthcare pyramid in most Countries and are considered as leaders in establishing best practices in various domains. Similarly, in digital healthcare, other hospitals and institutes look forward to such medical universities to be the leaders in adoption and standardization of digital health platforms. Further, medical universities also seem to be the most appropriate starting point to equip future physicians with the skills, knowledge, and attitudes to leverage digital health technology in their clinical practice [5]. However, the piece meal adoption of digital health systems in leading medical universities, has led to a mismatch between the envisaged impact of digital services therein [6, 7]. Also, despite the benefits and potential for revolutionizing healthcare delivery, no practical guidance exists for implementation of digital transformation projects in hospitals [8].

Healthcare is a complex domain with various processes and systems & large and diverse data that need to be integrated. Many hospitals lack a comprehensive digital strategy, technical expertise and sufficient qualified personnel to implement digital initiatives. They also do not fully understand the potential benefits and challenges of digital transformation. Further, digital transformation is misconstrued as only introducing set of applications or by bringing digitization in few aspects of hospital functioning. This leads to solutions working in silos and in turn creates new challenges of interoperability where various digital systems are unable to communicate or share data with each other even within the same hospital. Lack of detailed technical factors with focus on security and confidentiality has been highlighted as a major obstacle towards successful implementation of digital transformation [9, 10]. This problem is further compounded with the out-dated and inflexible legacy systems which are deployed in a lot of hospitals and thus struggle to integrate new digital technologies with existing systems and data, leading to duplicated or conflicting information. Further, Hospital information systems (HIS) store and process a vast amount of sensitive patient information, making them a prime target for cyber-attacks [11]. Ill-defined access controls may also lead to interception of communication, such as email or network traffic, can put sensitive patient information at risk of exposure. Lastly, digital transformation initiatives in hospitals very often ignore change management, as involving significant changes to existing processes and ways of working can lead to resistance and adoption issues among senior healthcare professionals. Very little evaluation of information systems and its technical requirements has been done in existing literature, with focus on either few domains or on a small user sample in single setting. Further, there is lack of literature identifying the technical requirements and system integrity of Information systems which can be deployed [12]. There is also very little information available to medical university and hospital administrators on hardware, software, network analysis, cyber security, etc. to enable them plan comprehensive implementation of digital systems. With the push towards a large-scale digital adoption in the country in the wake of Ayushman Bharat Digital Mission (ABDM), it becomes crucial to assess the gaps in the digital health journey of medical universities. Hence, this study was done at the apex medical university in India, as the findings herein shall be relevant for similar other institutes as well and shall help prepare a road map for digital health transformation for other medical universities and hospitals as well [13, 14, 15- 16]. Therefore, it is of interest to recommend a framework for the holistic adoption of digital health solutions in medical universities and tertiary care hospitals, in sync with the futuristic trends in IT Governance, network, software and hosting and cyber security.

## Methods:

The objective was to map the processes and IT systems at an apex medical university in India (henceforth referred to as 'Institute'), conduct a gap analysis and based on that create a template which can be used to transform an apex medical centre as a state of art medical university equipped with modern digital solutions and create a seamless technological experience for researchers and patients. This was aimed to improve efficiency and effectiveness of medical education and research, operations, patient care and availability of quality information to the decision makers. We propose this model with a potential to be used as a framework for large medical universities across the world which are aiming to undergo digital transformation. The study was conducted by a team of experts, to evaluate the systems and processes at the Institute and spanned over 120-man months. Several rounds of interactions were held with over thousands of stakeholders and on ground assessment spreading across 8 months was also done. Further, the study also included feedback from 41 leading international technology and consulting firms, which were invited for industry consultations (IC) to exchange information, knowledge and experience from industry perspective for providing innovative and advanced solutions for the design, development, implementation and maintenance of the proposed model. The objective of the study was to assess the readiness of existing infrastructure of the institute to handle digital transformation as well as to identify key features which would be essential in on-going the transformation journey.

## Results:

Based on multiple inputs received from stakeholders and on ground assessment, it was found that, despite the Institute being in existence for over half a century and numerous IT solutions being procured in the past 30 years, it was observed that majority of the patient care, research and office based functions are not digitalized. Multiple IT solutions deployed across the Institute, displayed different levels of maturity due to their installation being done in silos over many years. Accordingly, a digital health maturity assessment of the IT systems at the Institute was done. This was followed by thematic analysis of the collected data from different stakeholders was conducted. The data was categorised in eight broad categories and was further divided into sub-themes. The key findings against each theme have been described in Table 1 (see PDF). Based on these findings, discussions from stakeholders and industry consultants, we propose a 5-pillar model for implementing large scale information systems in medical universities and hospitals around the world.

## The 5 pillar model (Figure 1 - see PDF):

The 5-pillar model as proposed by our study details the process based on several discussions and feedback from various stakeholders. The primary goal of this model is to assure that the digital transformation generates institute value and to mitigate the risks that are associated with digitization. This can be done by implementing an organizational/Institute-wide structure with well-defined roles for the responsibility of information, business processes, applications and IT infrastructure. This also comprises of the combination of processes and structures implemented by top management to inform, direct, manage and monitor activities of the Institute towards the achievement of successful information systems.

## Pillar 1: Reliable network:

Our study highlighted the challenges which medical institutes globally face due to the legacy computer network which are built over the years which has outlived its defined life and original design. Hence, it is imperative that the legacy network be revamped and upgraded to meet the current requirement as well as be expandable to meet future requirements. It is also vital that the upgrade of the network should result in a resilient and managed network.

## Pillar 2: New-age secure application:

The study pointed the need to have state of art applications as very often institutes outlive the current applications and are in need of a new-gen application ecosystem with micro-services architecture, mobile-first approach, open and secure APIs which are also able to function without dependence on internet connectivity. This was prominently highlighted in the industry consultations as a lot of new applications have emerged in the recent years based on the recent advances in technology.

## Pillar 3: Reliable hosting (Data centre and disaster recovery):

One of the key takeaways from the survey was about the apprehensions amongst users regarding the speed of accessing various modules. The delay and time lags often create an undue pressure on users, which discourages uniform adoption of digital technologies. From the discussions between key stakeholders, this is partly attributed to traditional IT infrastructure design which often runs on unreliable networks. A major disadvantage with such systems is that it can lead to potential shutdown of applications and lead to disruption of services which can severely affect patient care. Also, there is a risk of data loss or data theft, which can also have potential legal complication. Further, the discussions highlighted the need to shift to cloud-architecture design which comes with a host of benefits and align with today's ever-increasing workloads. It is also imperative that institutes adopt a forward-looking vision to implement a cloud-infrastructure model on its own premises. Our model proposes that the hosting should fit hand-in- glove with data privacy laws of Digital Personal Data Protection Act, 2023 as well as help reap the benefits of traditional IT infrastructure with cloud-optimized architecture.

## Pillar 4: Robust security:

Considering the increasing number of cyber-attacks occurring in today's world, it is critical that medical universities adopt campus-wide security measures to protect its digital assets. As hospitals are involved in the management of the personal information of patients, it is extremely critical that they comply with all the necessary national/international government guidelines and secure its internal and external access to its network, secure its endpoint devices like desktops, laptops, wi-fi access points, secure its network switches and servers. The discussions proposed that a central office should be established with information security policy and procedures, review and audit mechanisms. In addition, the endpoints in the network should be monitored 24 x 7 via a managed Security Operations Centre (SOC) and threats identified should be mitigated and corrective actions should be implemented to prevent future attacks.

## Pillar 5: IT Governance framework:

Our study highlighted that there is very little focus on IT governance in medical institutes. Historically, academic institutions have not felt the need to have a robust IT policy due to non-alignment of application with the larger vision of the institute. Our findings also focused specifically on the implementation of digital systems, their performance and managing risk. The study highlighted that the governance framework creates IT alignment to ensure that projects and processes are working in sync and are contributing to long-term success of the academic institute. Further, this alignment also entails addressing the business risk associated with the use, ownership, operation, involvement, influence and adoption of information technology within an organization. The industry consultations highlighted the need for Risk Management where IT business risk which consists of "IT-related" events and can potentially impact the smooth functioning of the institute. The study also highlighted the need to have appropriate capabilities to execute the strategic plan and ensure that sufficient, appropriate and effective resources are provided. Lastly the framework focussed on performance management to measure all activities and resources consumed that lead towards achieving strategic outcomes.

## Discussion:

It is crucial that undertaking a digital transformation requires careful planning and execution, as well as on-going monitoring and improvement. The study highlighted that the planning should start by defining the requirements for the institute, including the needs and goals of different stakeholders, such as healthcare providers, patients and administrators. This should also include assessment of the existing systems and processes in place to identify areas for improvement. This was evident from the positive participation of many stakeholders who participated in the survey, conducted as a part of the study. The discussions also highlighted the need to develop a comprehensive project plan that outlines the goals, timeline, budget and resource requirements for the digital implementation and identify areas for improvement and ensuring that it is meeting the needs of the organization. Our study highlighted the importance of guiding principles before undertaking digital transformation of academic medical institutions. The challenges faced by institutions, along with its readiness and its road map for the future, need to be factored in before undertaking a transformation. The findings from the digital maturity assessment conducted highlighted the challenges faced due to partially managed IT infrastructure, incomplete adoption of technologies, lack of data privacy and access protocols, partial implementation of information sharing, inadequately utilised data and lack of adequately trained manpower. It is evident that user experience in a lot of academic institutions is not satisfactory due to poorly utilised digital technology. Also, as studies have shown, the digital transformation of institutions can be leveraged to improve not just patient care but also the overall functioning of the institute.

Our study also highlighted the need for having a framework which can guide institutions to undertake transformation exercise at such scale. Based on the findings of our study, we have proposed a model for implementing the transformation that will help ensure standardization, efficient development, integration, scalability and security, all of which are critical factors in delivering effective and efficient patient care. This is a first of kind study which provide a standardized approach to digital transformation, ensuring consistency and uniformity across different departments and facilities within a hospital. It also provides a structure for the development of applications, allowing developers to focus on specific components and reducing the time and effort required to build a complete system. Further, it facilitates the integration of various components of an HMIS, such as electronic health records (EHRs), laboratory information systems (LIS) and radiology information systems (RIS), ensuring seamless data exchange and improved patient care. As it is often ignored, this helps in ensuring the scalable development of software systems, making it easier to add new features and capabilities as the needs of the hospital evolve as well as providing a secure platform for the storage and exchange of sensitive patient information, protecting it from unauthorized access and ensuring compliance with privacy regulations. As evident from our model for advanced information systems in medical universities, it can bring several advantages, including improved patient care. This can be achieved by providing real-time access to patient information as advanced information systems can help healthcare providers make more informed decisions, resulting in improved patient care.

Further, this can automate many manual processes, such as appointment scheduling and record keeping, increasing the efficiency of medical universities. There is also a need for enhanced collaboration by providing a centralized platform for sharing information where advanced information systems can facilitate collaboration between different departments and healthcare providers, leading to better coordination of care. The most important advantage of undertaking digital transformation in medical institutions is that they can empower patients by providing them with easy access to their health information and enabling them to participate in their own care. This also supports academic and research by providing access to large amounts of data, enabling medical researchers to gain new insights and advance their field. Medical Universities can also utilise this model to support the education of medical students by providing them with access to a wealth of information and enabling them to gain a deeper understanding of their field. This also validates the assumption that the integration of Digital Health Technology in medical university curriculum represents a crucial opportunity with regards to improving the quality of care and preparing doctors for the future of medical practice [5]. Lastly digital systems can provide real-time data and analytics, enabling administrators and decision-makers to make more informed decisions about the management and direction of the medical university.

## Conclusion:

The implementation of digital transformation in medical universities can bring significant benefits in terms of improved patient care, increased efficiency and enhanced research and education.
